# Effectiveness of Acupuncture Treatment on Chemotherapy-Induced Peripheral Neuropathy: A Pilot, Randomized, Assessor-Blinded, Controlled Trial

**DOI:** 10.1155/2020/2504674

**Published:** 2020-06-29

**Authors:** Somayeh Iravani, Amir Hooman Kazemi Motlagh, Seyede Zahra Emami Razavi, Farhad Shahi, Jing Wang, Li Hou, Wenjun Sun, Mohammad Reza Afshari Fard, Mahdi Aghili, Mehrdad Karimi, Hossein Rezaeizadeh, Baixiao Zhao

**Affiliations:** ^1^School of Acupuncture-Moxibustion and Tuina, Beijing University of Chinese Medicine, Beijing 100029, China; ^2^School of Persian Medicine, Tehran University of Medical Science, Tehran, Iran; ^3^Department of Physical Medicine and Rehabilitation, Imam Khomeini Hospital Complex, Tehran University of Medical Science, Tehran, Iran; ^4^Department of Hematology and Medical Oncology, Imam Khomeini Hospital Complex, Cancer Institute Research Center, Tehran University of Medical Science, Tehran, Iran; ^5^Department of Hematology and Oncology, Dongzhimen Hospital, Beijing University of Chinese Medicine, Beijing 100700, China; ^6^Department of Neurology, 3^rd^ Affiliated Hospital, Beijing University of Chinese Medicine, Beijing 100029, China; ^7^Department of Radiation Oncology, Radiation Oncology Research Center, Tehran University of Medical Science, Tehran, Iran; ^8^School of Traditional Chinese Medicine, Beijing University of Chinese Medicine, Beijing 100029, China

## Abstract

**Objective:**

This pilot study aims to evaluate the effectiveness and safety of acupuncture in the treatment of chemotherapy-induced peripheral neuropathy (CIPN).

**Methods:**

This study was a pilot randomized controlled trial, which was conducted with cooperation between Beijing University of Chinese Medicine (BUCM), China, and Tehran University of Medical Science (TUMS), Iran. Forty participants with CIPN were randomly assigned (1 : 1) to receive twelve sessions of acupuncture (20 minutes each session over 4 weeks) or take one 300 mg tablet of vitamin B1 and three 300 mg capsules of gabapentin per day for 4 weeks, after which both groups were followed up for 4 weeks. The primary endpoint was CIPN symptom severity measured by the Numerical Rating Scale (NRS). The secondary endpoints included sensory neuropathy grade evaluated by the National Cancer Institute-Common Toxicity Criteria for Adverse Events (NCI-CTCAE), neurophysiological assessment of CIPN by the nerve conduction study (NCS), and the patient overall satisfaction with treatment. Safety was assessed at each visit.

**Results:**

The NRS and NCI-CTCAE sensory neuropathy grading scales decreased significantly over time in both groups (both *P* < 0.001), with a significantly higher reduction in the acupuncture group (*P* < 0.001 and *P* = 0.03, respectively). In addition, the acupuncture group showed a higher overall satisfaction with the treatment at the end of treatment and after 4 weeks follow-up, in comparison with the vit B1 and gabapentin group (*P* = 0.01 and *P* = 0.001, respectively). The NCS (except for the latency of the sural nerve) in the acupuncture group improved significantly (*P* < 0.05), while improvement in the vit B1 and gabapentin group was not observed (*P* > 0.05).

**Conclusion:**

Our study revealed that acupuncture, as a kind of traditional Chinese therapeutic method, is significantly effective and safe in the treatment of CIPN. Moreover, acupuncture is more effective than using vitamin B1 and gabapentin as the conventional treatment. *Trial registration.* This trial is registered with the Iranian Registry of Clinical Trials (IRCT20190615043900N1).

## 1. Introduction

Chemotherapy-induced peripheral neuropathy (CIPN) is one of the most common dose limiting adverse events of chemotherapeutic agents and is described as an injury to the peripheral nervous system due to neurotoxic chemotherapeutic agents [1] such as platinum compounds, taxanes, vinca alkaloids, bortezomib, and thalidomide [1, 2]. A systematic review and meta-analysis of 31 studies (4179 patients) investigated epidemiological measures of CIPN and established the prevalence of CIPN at 68.1% in the first month after chemotherapy, 60% at the third month, and 30% at 6 months or later [3], although this prevalence is influenced by duration of therapy, type of chemotherapy regimens, and assessment methods [4].

Most symptoms of CIPN are sensory and include numbness, tingling, and pain with typical distal symptoms occurring symmetrically in a glove-and-stocking distribution. Motor and autonomic neuropathies occur less frequently [5].

The pathophysiological mechanism of CIPN has not been totally discovered. Usually, neurotoxic drugs can induce an axonal poly neuropathy in different ways, such as by damaging microtubules, interfering with microtubule-related axonal transport, causing a disability of the mitochondria, making changes in the release of pain mediators such as growth factors, cytokines, and ion channels, and also with cytotoxic effects on DNA [5]. CIPN is also a big challenge for Traditional Chinese Medicine (TCM) oncologists, because it was not mentioned in classic TCM books [6]. TCM oncologists have classified it under the category of *Bi* syndrome [6, 7] and also classified it as *Bi* syndrome and *Wei* syndrome [8]. Pathogenesis of CIPN, according to TCM, is deficiency of qi and blood, qi stagnation, and blood stasis, which leads to the malnourishment of tendons and vessels, and stasis in channels and collaterals [6].

Although chemotherapy-induced nausea, vomiting, and neutropenia have been treated with the improvements in cancer supportive care, the treatment of CIPN still remains a challenge [9]. CIPN has two significant aspects, one being that it can affect the quality of life of cancer survivors, and second that it can also lead to dose reduction, early termination to less effective agents, or even discontinuation of treatment [10]. Several pharmacological agents such as tricyclic antidepressant (TCA), selective serotonin norepinephrine reuptake inhibitors (SSNRI), pregabalin, and gabapentin are recommended as the first-line of treatment for CIPN. However, at present, no ideal therapeutic agents are available for the prevention or even treatment of CIPN [11].

Acupuncture as a kind of traditional Chinese therapeutic method has been used as an effective and safe treatment for chemotherapy-induced nausea and vomiting [12], fatigue [13, 14], cancer related pain [15], lymphedema [16], anxiety, depression, and insomnia [17, 18]. Moreover, it has also been used for the treatment of neuropathic pain such as postherpetic neuralgia [19], diabetic peripheral neuropathy [20–23], and HIV related neuropathy [24]. The possible beneficial effect of acupuncture on the treatment of CIPN has been increasingly studied in recent years with some research [25–28]; however, the available evidence for its efficacy is inconclusive. A systematic review and meta-analysis [29] revealed that acupuncture can effectively relieve CIPN pain and improve quality of life, whereas a recent randomized controlled pilot study [30] deemed the clinical significance of acupuncture unclear. Moreover, the treatment of CIPN is often a significant problem in the management of patients with cancer. Although there are different therapeutic methods for peripheral neuropathy due to chemotherapy, their effect, application mode, and availability are different. For these reasons, randomized controlled trials are needed to investigate the therapeutic effectiveness of acupuncture in CIPN to improve the care of many patients who are suffering from CIPN. The aim of this current study is to evaluate the effectiveness and safety of acupuncture in the treatment of CIPN. We hope the result of this pilot randomized controlled trial will provide basis for planning large randomized controlled trials in the future.

## 2. Materials and Methods

### 2.1. Design and Setting

This study is a pilot randomized controlled trial, which was conducted with cooperation between Beijing University of Chinese Medicine (BUCM), China, and Tehran University of Medical Science (TUMS), Iran, from June 2017 to December 2018. The study protocol was approved by the Ethics Committee Board of Beijing University of Chinese Medicine (2017BZHYLL0317) and also of Tehran University of Medical Science (IR.TUMS.VCR.REC.1397.362). This study was performed according to the guidelines and principles of the Declaration of Helsinki. Informed written consent was obtained from all of participants before randomization.

### 2.2. Participants

Patients with CIPN were recruited in the Imam Khomeini Hospital in Iran and the Dongzhimen Hospital in China. After the initial screening evaluation, patients were enrolled in the study if they met all of the inclusion criteria and were excluded if they had any of the exclusion criteria.

#### 2.2.1. Sample Size

When we designed this study, there were few RCTs that have assessed the effectiveness of acupuncture on CIPN. There was no previous study to base a sample size on. Therefore, we aimed to detect a clinically important difference of 2 points in the Numerical Rating Scale (NRS). This was extrapolated from the clinical trials for multiple conditions, that is, diabetic neuropathy [31]. We calculated that a minimum sample size of 16 participants per group was necessary to provide 90% power to detect a clinically important difference of 2 points in the NRS between groups, assuming a standard deviation of 1.7 [2] and a 2-sided significance level of 5%. To account for a 20% drop-out rate, the sample size was increased to 20 participants in each group.

#### 2.2.2. Inclusion Criteria

Patients were included if they (1) were aged between 18 and 70 years, (2) have received neurotoxic chemotherapy (at least one complete course), (3) have experienced symptoms of chemotherapy-induced peripheral neuropathy for more than three months after the completion of chemotherapy, (4) had scores ≥4 on 10 for tingling, numbness, or pain on the Numerical Rating Scale (NRS), and (5) accepted and signed an informed consent form, and (6) did not use medications such as tricyclic antidepressants (TCA), calcium channel blockers, and membrane stabilizing drugs for the prevention or treatment of the neuropathy for at least one month before enrollment.

#### 2.2.3. Exclusion Criteria

Exclusion criteria included (1) having a history of disease that causes neuropathy, such as diabetes, multiple sclerosis, HIV, and Parkinson's disease, (2) the presence of peripheral neuropathy or history of peripheral neuropathy due to any cause excluding chemotherapy, (3) alcohol abuse, (4) pregnancy, (5) psychological disease, and (6) severe dysfunction of the heart, kidneys, or liver.

### 2.3. Randomization

Eligible participants were randomized in a ratio of 1 : 1 to either the acupuncture group or control group. A block randomization list was created by the ‘blockrand' package in R software (version 3.3.3), based on *n* = 40 participants and two treatments. The allocation sequence was concealed from the researchers in sealed, opaque, and sequentially numbered envelopes. After the researcher had assessed eligibility, obtained the participant's consent, and completed all baseline evaluations of the participants, corresponding envelopes were opened and treatment allocation was revealed (randomization was performed one time, based on 40 participants in 2 centers).

### 2.4. Blinding

In acupuncture research, it is not possible to blind the practitioner, and the participants in this study were also aware of the type of treatment because one group received acupuncture and the other group received pharmacological medication, and it was not feasible to conceal allocation from participants. However, treatment and evaluation was performed independently. Subjective and objective evaluations and statistical analyses were performed by blinded specialists, who were not aware of the allocation of participants.

### 2.5. Intervention

#### 2.5.1. Acupuncture Group

Acupuncture treatment was implemented three times per week for four weeks. According to the literature reviews [32–36] and clinical experiences of a responsible researcher, two groups of points were used including local points and general points: Qihai (CV 6), Baihui (GV 20), Bilateral Zusanli (ST 36), Sanyinjiao (SP 6), Hegu (LI 4), Quchi (LI 11), and Taichong (LR 3) as the general points and bilateral Bafeng (EX-LE 10) and Baxie (EX-UE 9) as the local points. Patients with CIPN symptoms in the lower extremities were treated with only Bafeng (EX-LE 10), while patients with CIPN symptoms in the upper extremities were treated with only Baxie (EX-UE 9). Patients with CIPN symptoms in both the upper and lower extremities were treated with a combination of these two points. Additional individualized points were used, if needed, according to patient symptoms, including Tianshu (ST 25), Waiguan (SJ 5) and Zhaohai (KI 6) for constipation, Neiguan (PC 6) and Zhongwan (CV 12) for vomiting, and Sishencong (EX-HN1) and Shenmen (HE 7) for insomnia. When applying general points, a reinforcing technique was used at Qihai (CV 6), Zusanli (ST 36), Sanyinjiao (SP 6), and Baihui (GV 20), while a reducing technique was applied at Hegu (LI 4) and Quchi (LI 11), and an even technique was implemented at Taichong (LR 3). For local points, only a reducing technique was used. After using alcohol for local skin sterilization, disposable sterilized filiform needles (0.25 × 0.40 mm; Zhongyan Taihe, Beijing Zhongyan Taihe Medical Instruments center, Beijing, China) were inserted perpendicularly at the depth of 10–15 mm in general points and 5–7 mm in local points, with proper needling manipulation to induce “de qi” (the arrival of qi). After achieving de qi, the needles were retained for 20 minutes.

The acupuncture treatments were performed by a practitioner of western medicine, who completed 2 years of full time training in acupuncture, moxibustion, and tuina. The supervisor, a professor with more than 30 years of clinical experience, monitored the execution of treatment protocols throughout the study.

#### 2.5.2. Vit B1 and Gabapentin Group

In this group, the treatment consisted of one 300 mg tablet of vitamin B1 and three 300 mg capsules of gabapentin per day for four weeks.

### 2.6. Outcome Measures

To determine efficacy, patients were evaluated according to both subjective and objective outcome measurements. Subjective assessment was collected using the Numerical Rating Scale (NRS), National Cancer Institute-Common Toxicity Criteria for Adverse Events (NCI-CTCAE) sensory neuropathy grading scale, and patient report of overall satisfaction with the treatment, while objective assessment included the nerve conduction study (NCS). For safety, all patients in the acupuncture group were assessed for signs and/or reports of excessive bruising, local persistent pain, and evidence of bleeding after each acupuncture session, and all adverse events that were reported by patients and researchers were recorded in both groups and monitored until its resolution.

#### 2.6.1. Primary Outcome Measurement

The primary outcome was measured using the Numerical Rating Scale (NRS), which is frequently used for the assessment of symptom severity in CIPN, and it has some documented validity in cancer patients [37]. CIPN symptom severity was assessed by asking patients to rate their average neuropathic symptoms, such as tingling, numbness, and pain, on an 11-point scale over the course of a particular day (0: no symptoms and 10: the worst possible symptoms imaginable). Primary outcome measurement was performed at baseline, 2 and 4 weeks after starting treatment, as well as 4 weeks after the end of treatment (after 8 weeks).

#### 2.6.2. Secondary Outcome Measurement

The secondary outcome measures included the following.

*(1) National Cancer Institute-Common Terminology Criteria for Adverse Events (NCI-CTCAE) Sensory Neuropathy Grading Scale*. NCI-CTCAE v.4.03 is the standard classification and severity grading scale for adverse events and can be used in clinical trials, the treatment of cancer, and so on. It is a subjective, standardized method for the classification of CIPN severity that is easy and quick to use and has documented reliability and validity [38]. Severity of the peripheral sensory neuropathies was classified using a 5-point scale ranging from grade 1 to grade 5. It was performed at baseline, 2 and 4 weeks after starting treatment, as well as 4 weeks after the end of treatment (after 8 weeks).

*(2) Nerve Conduction Study (NCS)*. The NCS is a noninvasive, objective gold standard for the neurophysiological assessment of CIPN [39]. The bilateral NCS of the lower limbs was performed with a Medelec V, USA system in Iran, and a Nicolet Viking 4, USA system in China. Skin surface electrodes were used to record the motor and sensory nerve conduction. Motor conduction was studied in the bilateral peroneal and tibial nerves by recording the onset latency and the amplitude of compound motor action potentials (CMAP), as well as motor conduction velocity. Sensory conduction was tested in the bilateral sural nerves by measuring the onset latency and the amplitude of sensory nerve action potentials (SNAP), as well as sensory conduction velocity. NCS was carried out before and after treatment (after 4 weeks).

*(3) Patient Overall Satisfaction with Treatment*. The patients' overall satisfaction with treatment was measured on a four-point Likert-type scale, where patients rated their overall satisfaction with the treatment since the beginning of the study, including 1: not at all satisfied, 2: slightly satisfied, 3: moderately satisfied, and 4: completely satisfied. Patients were assessed with this scale at the end of treatment and after 4 weeks follow-up (after 8 weeks).

*(4) Safety Assessment*. All patients were additionally assessed for safety after each acupuncture session, and side effects occurring in either treatment group were recorded.

### 2.7. Statistical Analysis

Statistical analysis was performed with the use of SPSS (v20.0, IBM) and STATA (v11.0, SE) by the statistician, who was blinded from the groups allocation. Variables, which were measured at several different times, were assessed by using mixed between-within-subjects analysis of variance (ANOVA) or ordinal logistic regression, as appropriate. For other variables, paired samples or nonparametric Wilcoxon tests were used for the assessment of data in each group and independent samples or nonparametric Mann–Whitney *U* tests were used for the evaluation of data between two groups. A *P* value lower than 0.05 was considered statistically significant in this study.

## 3. Results

**Figure 1:**
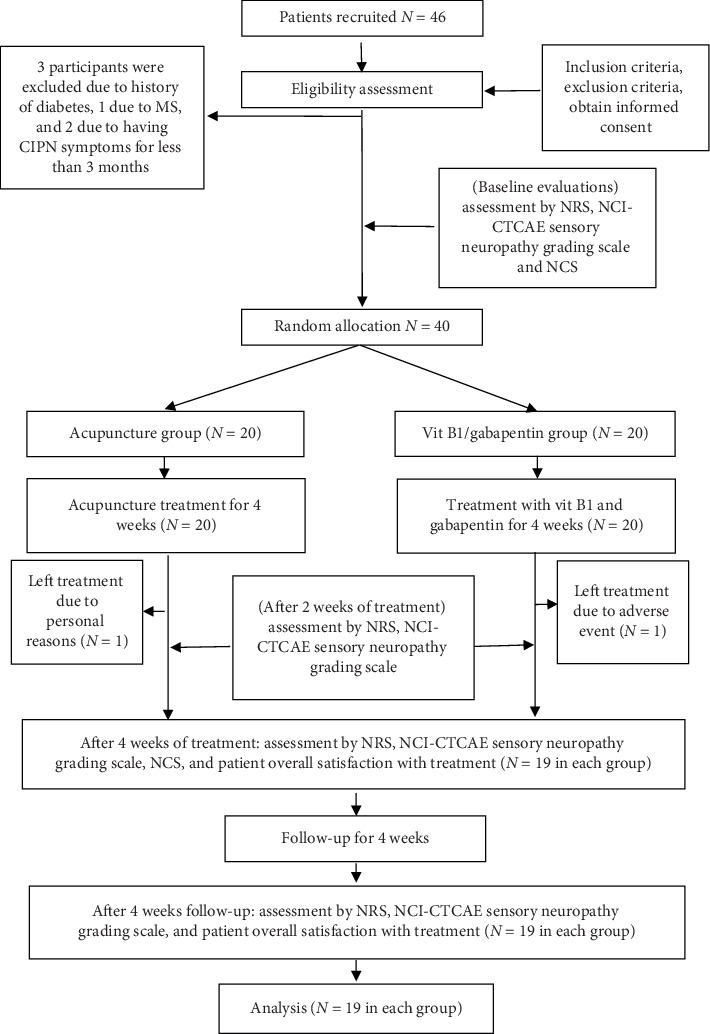
Flowchart of the study. MS: multiple sclerosis, CIPN: chemotherapy-induced peripheral neuropathy, NRS: Numerical Rating Scale, NCS: nerve conduction study, NCI-CTCAE: National Cancer Institute-Common Terminology Criteria for Adverse Events.

A total of 46 patients were screened in this study and 3 participants were excluded due to a history of diabetes, 1 due to multiple sclerosis (MS) and 2 due to having symptoms of CIPN for less than 3 months. Finally, 40 patients, who met the eligibility criteria, were enrolled in this study (two-thirds of the patients were enrolled in Iran and one-third in China). Two patients stopped treatment during this study: one patient in the vit B1 and gabapentin group left the study due to the side effects of gabapentin, including somnolence and dizziness. In addition, one patient in the acupuncture group refused to continue treatment for personal reasons. Thus, data of the 38 patients who completed the treatment and evaluation was included in the analysis (Figure 1).

### 3.1. Basic Characteristics

Among the 38 patients who completed the study, there were 23 (60.5%) female and 15 (39.5%) male, with the mean age of 57.95 ± 10.39 years in the acupuncture group, and 58.79 ± 8.36 years in the vit B1 and gabapentin group. According to the type of cancer, 18 (47.4%) patients had breast cancer, 16 (42.2%) had colorectal cancer, 1 (2.6%) had lung cancer, 1 (2.6%) had ovarian cancer, and 2 (5.3%) had prostate cancer. Twenty (52.7%) patients had been treated with taxanes, 16 (42.1%) with platinum compounds, and 2 (5.3%) with a combination of taxanes and platinum compounds. The mean duration of neuropathy was 7.32 ± 6.17 months and the mean number of chemotherapy courses was 7.50 ± 2.30. The most common symptoms of CIPN were paresthesia (100%), numbness (94.7%), pain (71.1%), and subjective impairment in walking (13.2%).

The participants' baseline demographic and clinical characteristics were similar between groups (Table 1).

### 3.2. Response to the Treatment

#### 3.2.1. Primary Outcome

**Figure 2:**
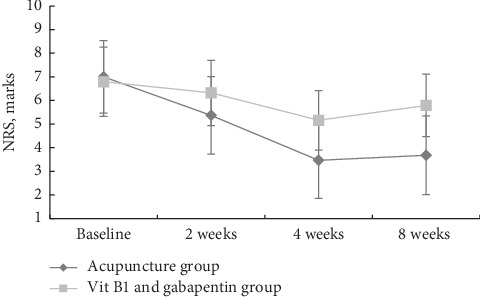
Comparison of NRS at baseline, after 2 and 4 weeks of treatment and 4 weeks of follow-up (8 weeks) between the 2 groups (all values are mean ± SD).

The initial NRS scores in the acupuncture and vitB1/gabapentin groups were 7.00 ± 1.53 and 6.79 ± 1.47, respectively. Acupuncture treatment reduced the NRS gradually by 3.32 ± 1.73 points during the 8 weeks of treatment and follow-up, whereas that of the vit B1 and gabapentin decreased by 1 ± 1.11 points. The test of within-subjects effects of mixed between-within-subjects analysis of variance (ANOVA) revealed that the NRS scale at baseline, after 2 and 4 weeks of treatment and 4 weeks follow-up (8 weeks), in both groups were decreased in a time-dependent manner (Wilks' lambda = 0 .18, partial eta squared = 0.82, and *P* < 0.001), and in time and treatment interaction (Wilks' lambda = 0.49, partial eta squared = 0.51, and *P* < 0.001), suggesting significant differences between NRS changes over time across the two groups (Figure 2). Furthermore, there was a significant difference in NRS scores between the two groups (partial eta squared = 0.16 and *P* = 0.01) and pairwise comparisons (post hoc analysis) showed significant differences in NRS scale between the two groups after 2 and 4 weeks of treatment (mean difference (MD) = −1.05, 95% confidence interval (CI) = −1.28 to −0.83, *P* < 0.001 and MD = −2.58, 95% CI = −3.04 to −2.12, *P* < 0.001, respectively) and was maintained after the 4 weeks of follow-up (MD = −2.16, 95% CI = −2.64 to −1.68, *P* < 0.001), in favor of the acupuncture treatment (Table 2).

#### 3.2.2. Secondary Outcome

*(1) National Cancer Institute-Common Terminology Criteria for Adverse Events (NCI-CTCAE) Sensory Neuropathy Grading Scale*. After 8 weeks of treatment and follow-up, improvement in sensory neuropathy, based on the NCI-CTCAE v.4.03 scale (decrease in neuropathy by at least one grade), was found in 13/19 (68.4%) and 3/19 (15.8%) of patients in the acupuncture group and vit B1/gabapentin group, respectively. Moreover, in 1/19 (5.3%) and 0/19 (0%) of patients receiving acupuncture and vitB1/gabapentin neuropathy improved from grade 3 to grade 1, respectively (Table 3). The test of ordinal logistic regression showed that the grade of sensory neuropathy based on the NCI-CTCAE scale in both groups decreased over time (coefficient = −1.03, *Z* = −4.19, *P* < 0.001). Furthermore, there was a significant interaction between treatment and time (Chi_2_ = 12.16 and *P* = 0.03), suggesting significant differences between NCI-CTCAE scale changes over time across the two groups, in favor of acupuncture treatment.

*(2) Nerve Conduction Study*. The result of statistics in Table 4 showed that the NCS (except latency of the sural nerve) in the acupuncture group improved significantly (*P* < 0.05), while improvement in the vit B1 and gabapentin group was not observed (*P* > 0.05). Compared with the vit B1 and gabapentin group, the acupuncture group showed a significant increase in the amplitude of bilateral sural (MD = 2.7, 95% CI = 1.11 to 4.30, *P*=0.002) and tibial nerves (MD = 1.14, 95% CI = 0.51 to 1.77, *P*=0.001) after 4 weeks of treatment, but not in the peroneal nerve (MD = 0.81, 95% CI = 0.006 to 1.62, *P*=0.052). Moreover, acupuncture led to a significant increase in the motor conduction velocity (MCV) of the bilateral peroneal (MD = 2.63, 95% CI = 0.32 to 4.95, *P*=0.03) and tibial nerves (MD = 2.01, 95% CI = 0.56 to 3.46, *P*=0.008), as compared with the vit B1 and gabapentin group after 4 weeks of treatment, but not in the sensory conduction velocity (SCV) of the bilateral sural nerve (MD = 4.53, 95% CI = −4.00 to 13.05, *P*=0.29). In contrast, there were no significant differences between the 2 groups in onset latency of the bilateral sural (MD = −0.21, 95% CI = −0.85 to 0.42, *P*=0.50), peroneal (MD = −0.22, 95% CI = −0.49 to 0.05, *P*=0.11), and tibial (MD = −0.28, 95% CI = −0.69 to 0.14, *P*=0.19) nerves after 4 weeks of treatment.

*(3) Patients Overall Satisfaction with Treatment*. Compared with the vit B1 and gabapentin group, the acupuncture group showed a higher satisfaction with the treatment at the end of treatment and after 4weeks of follow-up (*P* = 0.01 and *P* = 0.001, respectively), as shown in Table 5. Moreover, there was no significant reduction in patient overall satisfaction with treatment after 4 weeks of follow-up compared to after treatment in the acupuncture group (*P* = 0.18), while patient overall satisfaction with treatment significantly decreased after 4 weeks of follow-up compared to after treatment in the vit B1 and gabapentin group (*P* = 0.01).

*(4) Safety Assessment*. There were no adverse events related to acupuncture treatment during this study. No excessive bruising, local persistent pain, or evidence of bleeding was reported, while one patient in the vit B1 and gabapentin group left the study due to side effects from gabapentin, including somnolence and dizziness.

## 4. Discussion

In this study, we described the effectiveness and safety of acupuncture for the treatment of CIPN. After 8 weeks of treatment and follow-up, the acupuncture group showed a greater reduction in the NRS than the vit B1 and gabapentin group (3.32 ± 1.73 versus 1 ± 1.11). This is further supported by the clinician-rated NCI-CTCAE where significant improvements were also seen (68.4% versus 15.8%). Moreover, the NCS (except for the latency of the sural nerve) was improved best in the acupuncture group and no adverse events were observed, showing that acupuncture is a safe and effective way to treat CIPN. These results are consistent with those from available small-scale pilot/feasibility trials or uncontrolled trials and case series that found acupuncture is effective in the treatment of CIPN [25, 33, 34, 40] and studies of Schroder et al. [36] and Han et al. [26] which showed improvement in the NCS after acupuncture treatment for CIPN. In our study, the mean difference between the two groups in the NRS was 2.16 points, which compares favorably to the clinically important difference of 2 points reported in the past studies [31]. The NCI-CTCAE also showed statistically significant improvement in favor of the acupuncture group, although there is no established clinically important difference value for NCI-CTCAE. In addition to the magnitude of improvement, several other factors should also be considered when determining clinical significance, such as patient adherence, safety, and tolerability [41]. Consistent with our results, several recently published studies showed that acupuncture is safe and well tolerated [25, 26, 29, 30]. Therefore, after considering these factors in addition to the improvement of the NRS, NCI-CTCAE scores, and NCS, our study provided encouraging results regarding the use of acupuncture for the treatment of CIPN.

The mechanism by which acupuncture may affect CIPN and improve NCS is not fully understood. However, Litscher et al. [42] showed that acupuncture can increase the circulation of blood in the extremities, which causes more circulation of blood to the vasa nervorum and related capillary beds nourishing the neurons and may contribute to nerve repair with measurable improvement of axons or myelin sheaths. In addition, the analgesic effect of acupuncture has been shown in animal studies, and the symptomatic effect of acupuncture may be due to the stimulation of nerves that innervate muscles, thus leading to a release of neurotransmitters such as endorphin and encephalin, which regulate the function of the spinal cord, midbrain, and hypothalamo-hypophyseal pathways [43].

Several different acupuncture concepts have been applied in the treatment of CIPN; however, in most previous studies that reported acupuncture as an effective treatment for CIPN, local points like Bafeng (EX-LE10) and Baxie (EX-UE9) were used [25, 32, 33, 35, 36], similar to our study. From the biomedical point of view, choosing to use local points is appropriate for the treatment of CIPN. Acupuncture has been shown to increase blood circulation locally [44, 45]. Therefore, local acupuncture may heal local structures by transporting oxygen and nutrients and by eliminating metabolic waste. In addition, studies propose that the therapeutic effects of acupuncture may be communicated through its interaction with connective tissue, especially by the stimulation of contraction, which has been shown to improve wound healing and tissue regeneration [46]. Considering how connective tissue creates a vast system surrounding muscle, organs, nerve, blood vessels, and lymphatics, acupuncture may cause local nerve healing through nearby connective tissue contraction.

On the basis of Traditional Chinese Medicine theory, the pathogenesis of CIPN includes deficiency of qi and blood, qi stagnation, and blood stasis, which lead to the malnourishment of tendons and vessels, and stasis in channels and collaterals [6]. We describe our successful treatment of CIPN with acupuncture according to our approach that tonifies body qi and blood and directs their flow to the extremities. The base and general therapeutic principles in this study included dredging channels and collaterals, activating the circulation of qi and blood to remove blood stasis, tonifying qi and nourishing blood, supplementing the liver and kidney, calming the mind, and regulating the *Shen*. In addition, according to the layer analysis method of the Yellow Emperor's Inner Classic text, diseases at the skin layer present symptoms such as numbness, insensitivity, and temperature changes on the skin layer. The choice of appropriate acupuncture techniques for different layers is crucial for successful treatment of patients. Needling techniques such as direct subcutaneous needling were primarily used for treating diseases at the skin layer [47]. Therefore, we used a shallow direct insertion of needles at Bafeng (EX-LE10) and Baxie (EX-UE9) points.

In summary, as a pilot RCT, the results of this study gave a promising treatment option for the treatment of CIPN with little side effects. We set vit B1 and gabapentin, a conventional treatment, as the control in this study and used the NCS to evaluate the treatment, which is an objective and quantitative parameter of peripheral nerve functions. Compared to the previous studies, especially those performed outside of China, we pay more attention on the classical theory of acupuncture and clarified the needling techniques at different points. We conducted our research in Iran and China with patients of different ethnicities, which increased the generalizability of the findings. The main findings of this study may give a new point of view to physicians for the treatment of CIPN in the future.

Finally, this study also has some limitations. Firstly, the sample size of this study was small and no power analysis was performed which lowers the statistical power of this study. For a better judgment of our findings, the sample size of the study should be larger. Secondly, we did not have a control group without any treatment to determine whether the therapeutic effect was due to our treatment or because of time and natural process. Thirdly, we did not have a sham acupuncture group to eliminate possible acupuncture placebo effects and give more valid information about the efficacy of acupuncture. Lastly, a lack of long-term follow-up assessments was also a limitation. A longer duration of follow-ups is needed to reveal long-term beneficial effects of acupuncture in the treatment of CIPN.

## 5. Conclusion

Our study revealed that acupuncture as a kind of Traditional Chinese therapeutic method is significantly effective and safe in the treatment of CIPN. Moreover, acupuncture is more effective than using vitamin B1 and gabapentin as the conventional treatment. Further, large randomized controlled trials with a long-term follow-up are needed in the future to confirm the beneficial effect of acupuncture in the treatment of CIPN and to find whether the effects are long-lasting.

## Figures and Tables

**Table 1:** Comparison of participants' baseline demographic and clinical characteristics between two groups.

Characteristics	Acupuncture (*N* = 19)	Vit B1 and gabapentin (*N* = 19)	*P* value
Age, mean ± SD, years	57.95 (10.39)	58.79 (8.36)	0.79^*∗*^
Gender, no. (%)			0.74^*∗∗*^
Male	7 (36.8)	8 (42.1)	
Female	12 (63.2)	11 (57.9)	
Cancer type, no. (%)			0.91^*∗∗*^
Breast cancer	10 (52.6)	8 (42.1)	
Lung cancer	1 (5.3)	0	
Ovarian cancer	0	1 (5.3)	
Prostate cancer	1 (5.3)	1 (5.3)	
Colorectal cancer			
Colon	3 (15.8)	5 (26.3)	
Rectum	4 (21.1)	4 (21.1)	
Chemotherapy agents, no. (%)			0.93^*∗∗*^
Taxane	1 (5.3)	1 (5.3)	
Platinum compound	7 (36.8)	9 (47.4)	
Platinum compound-taxane	1 (5.3)	1 (5.3)	
Doxorubicin/cyclophosphamide-taxane	10 (52.6)	8 (42.1)	
Duration of neuropathy, mean ± SD, month	7.16 (7.06)	7.47 (5.32)	0.88^*∗*^
Number of chemotherapy courses, mean ± SD	7.58 (2.63)	7.42 (1.98)	0.84^*∗*^
Patients symptoms, no. (%)			
Pain			0.72^*∗∗*^
Yes	14 (73.7)	13 (68.4)	
No	5 (26.3)	6 (31.6)	
Numbness			
Yes	18 (94.7)	18 (94.7)	
No	1 (5.3)	1 (5.3)	
Paresthesia			
Yes	19 (100)	19 (100)	
No	0	0	
Subjective impairment in walking			0.63^*∗∗*^
Yes	3 (15.8)	2 (10.5)	
No	16 (84.2)	17 (89.5)	

^*∗*^ *P* values from between-group comparison using the independent samples test. ^*∗∗*^*P* values from between-group comparisons using the chi-square test.

**Table 2:** Comparison of changes of NRS within and between groups over time.

Variables	Time	Acupuncture group (*N* = 19)		Vit B1 and gabapentin group (*N* = 19)		Comparison between two groups	
		Mean (SD)	*P* value^*∗*^ (within-group effect)	Mean (SD)	*P* value^*∗*^ (within-group effect)	Difference (95% CI)	*P* value^*∗∗*^
NRS, marks	Baseline	7.00 (1.53)	<0.001	6.79 (1.47)	<0.001		
	2 weeks	5.37 (1.64)		6.32 (1.38)		−1.05 (−1.28 to −0.83)	<0.001
	4 weeks	3.47 (1.61)		5.16 (1.26)		−2.58 (−3.04 to −2.12)	<0.001
	8 weeks	3.68 (1.67)		5.79 (1.32)		−2.16 (−2.64 to −1.68)	<0.001

^*∗*^ *P* values from within-group comparison using mixed between-within-subjects analysis of variance (ANOVA). ^*∗∗*^*P* values from pairwise comparisons, adjustment for multiple comparison: least significant difference. NRS = Numerical Rating Scale.

**Table 3:** NCI-CTCAE sensory neuropathy grading scale over time according to group.

	Acupuncture group (*N* = 19)	Vit B1/gabapentin group (*N* = 19)
Baseline		
Grade 1	0	0
Grade 2	16 (84.2)	17 (89.5)
Grade 3	3 (15.8)	2 (10.5)

2 weeks		
Grade 1	5 (26.3)	1 (5.3)
Grade 2	12 (63.2)	16 (84.2)
Grade 3	2 (10.5)	2 (10.5)

4 weeks		
Grade 1	14 (73.7)	5 (26.3)
Grade 2	3 (15.8)	12 (63.20)
Grade 3	2 (10.5)	2 (10.5)

8 weeks		
Grade 1	13 (68.4)	3 (15.8)
Grade 2	4 (21.1)	14 (73.7)
Grade 3	2 (10.5)	2 (10.5)

NCI-CTCAE = National Cancer Institute-Common Terminology Criteria for Adverse Events. All values are no. (%).

**Table 4:** Comparison of changes of NCS within and between groups.

Variables	Time	Acupuncture group (*N* = 19)			Vit B1 and gabapentin group (*N* = 19)			Comparison between two groups	
		Mean (SD)	Mean diff.^a^(95% CI)	*P* value^*∗*^	Mean (SD)	Mean diff.^a^ (95% CI)	*P* value^*∗*^	Mean diff. (95% CI)	*P* value^*∗∗*^
*Sural nerve*									

Onset latency, ms	Baseline	2.58 (1.40)			2.51 (1.38)				
	4 weeks	2.37 (1.09)	−0.21 (−0.62 to 0.20)	0.30	2.51 (1.40)	0.004 (−0.51 to 0.52)	0.988	−0.21 (−0.85 to 0.42)	0.50

Amplitude, *μ*v	Baseline	3.56 (2.48)			3.62 (2.20)				
	4 weeks	6.62 (3.60)	3.06 (1.78 to 4.34)	<0.001	3.98 (2.49)	0.35 (−0.68 to 1.39)	0.482	2.7 (1.11 to 4.30)	0.002

SCV, m/s	Baseline	34.05 (18.46)			33.79 (18.29)				
	4 weeks	39.21 (17.72)	5.16 (0.41 to 9.90)	0.04	34.42 (18.61)	0.63 (−6.82 to 8.08)	0.861	4.53 (−4.00 to 13.05)	0.29

*Peroneal nerve*									

Onset latency	Baseline	3.57 (1.36)			3.50 (1.42)				
	4 weeks	3.37 (1.26)	−0.20 (−0.37 to −0.04)	0.02	3.51 (1.38)	0.02 (−0.20 to 0.24)	0.865	−0.22 (−0.49 to 0.05)	0.11

Amplitude	Baseline	3.00 (1.52)			3.23 (2.25)				
	4 weeks	3.82 (2.40)	0.82 (0.11 to 1.52)	0.03	3.24 (2.14)	0.005 (−0.44 to 0.45)	0.979	0.81 (0.006 to 1.62)	0.052

MCV	Baseline	36.57 (13.61)			37.02 (13.59)				
	4 weeks	39.45 (14.45)	2.87 (1.16 to 4.58)	0.002	37.26 (13.63)	0.24 (−1.44 to 1.93)	0.766	2.63 (0.32 to 4.95)	0.03

*Tibial nerve*									

Onset latency	Baseline	4.64 (1.31)			4.53 (1.89)				
	4 weeks	4.35 (1.25)	−0.29 (−0.57 to −0.01)	0.04	4.52 (1.82)	−0.01 (−0.34 to 0.32)	0.937	−0.28 (−0.69 to 0.14)	0.19

Amplitude	Baseline	5.63 (2.52)			5.75 (3.28)				
	4 weeks	6.80 (2.92)	1.17 (0.65 to 1.68)	<0.001	5.77 (2.92)	0.03 (−0.37 to 0.43)	0.891	1.14 (0.51 to 1.77)	0.001

MCV	Baseline	38.74 (10.07)			38.22 (13.99)				
	4 weeks	40.81 (10.54)	2.08 (0.89 to 3.26)	0.002	38.29 (13.90)	0.07 (−0.85 to 0.99)	0.878	2.01 (0.56 to 3.46)	0.008

^*∗*^ *P* values from within-group comparison using the paired samples test. ^*∗∗*^*P* values from between-group comparison using the independent samples test. ^a^Mean difference from baseline. NCS = nerve conduction study. SCV = sensory conduction velocity. MCV = motor conduction velocity.

**Table 5:** Comparison of patient overall satisfaction with treatment between two groups.

	After treatment (4 weeks)			After 4 weeks of follow-up (8 weeks)		
	Acupuncture group (*N* = 19)	Vit B1/gabapentin group (*N* = 19)	*P* value^*∗*^	Acupuncture group (*N* = 19)	Vit B1/gabapentin group (*N* = 19)	*P* value^*∗∗*^
Patient overall satisfaction with treatment, no. (%), marks						
Not at all satisfied	0	2 (10.5)	0.01	0	4 (21.1)	0.001
Slightly satisfied	4 (21.1)	10 (52.6)		5 (26.3)	11 (57.9)	
Moderately satisfied	5 (26.3)	3 (15.8)		5 (26.3)	2 (10.5)	
Completely satisfied	10 (52.6)	4 (21.1)		9 (47.4)	2 (10.5)	

^*∗*^ *P* value from between-group comparison after treatment (4 weeks) using Mann–Whitney *U* test. ^*∗∗*^*P* value from between-group comparison after 4 weeks of follow-up (8 weeks) using Mann–Whitney *U* test.

## Data Availability

The databases generated and/or analyzed during the current study are not publicly available as this was not included in the original ethics application. But the datasets are available from the corresponding author on reasonable request and approval from the research ethics committee of both universities.

## Abbreviations

CIPN: Chemotherapy-induced peripheral neuropathy
CMAP: Compound motor action potentials
MS: Multiple sclerosis
MCV: Motor conduction velocity
NRS: Numerical Rating Scale
NCI-CTCAE: National Cancer Institute-Common Toxicity Criteria for Adverse Events
NCS: Nerve conduction study
SSNRI: Selective serotonin norepinephrine reuptake inhibitors
SNAP: Sensory nerve action potentials
SCV: Sensory conduction velocity
TCM: Traditional Chinese Medicine
TCA: Tricyclic antidepressant.
